# Influence of cooking methods and storage time on colour, texture, and fatty acid profile of a novel fish burger for the prevention of cognitive decline

**DOI:** 10.1016/j.heliyon.2024.e27171

**Published:** 2024-02-28

**Authors:** Jorge Valentim, Cláudia Afonso, Romina Gomes, Ana Gomes-Bispo, José A.M. Prates, Narcisa M. Bandarra, Carlos Cardoso

**Affiliations:** a Faculty of Science, University of Lisbon, Campo Grande, 1749-016 Lisbon, Portugal; b Division of Aquaculture, Upgrading, and Bioprospection (DivAV), Portuguese Institute for the Sea and Atmosphere (IPMAIP), Avenida Alfredo Magalhães Ramalho, 6, 1495-165, Algés, Portugal; c CIIMAR, Interdisciplinary Centre of Marine and Environmental Research, University of Porto, Rua dos Bragas 289, 4050-123, Porto, Portugal; d MEtRICs/DCTB/NOVA, School of Science and Technology, NOVA University Lisbon, Caparica Campus, 2829-516, Almada, Portugal; e Centro de Investigação Interdisciplinar em Sanidade Animal (CIISA), Faculdade de Medicina Veterinária, Universidade de Lisboa, 1300-477, Lisbon, Portugal; f Laboratório Associado para Ciência Animal e Veterinária (AL4AnimalS), Faculdade de Medicina Veterinária, Universidade de Lisboa, 1300-477, Lisbon, Portugal

**Keywords:** Colour, Culinary treatment, Polyene index, *Scomber colias*, Storage stability, Texture

## Abstract

Western diets are poor in healthy n-3 polyunsaturated fatty acids (n-3 PUFA), namely eicosapentaenoic (EPA) and docosahexaenoic acid (DHA), iodine (I), and other nutrients that may protect against cognitive ageing. Given DHA richness in chub mackerel (*Scomber colias*), high vitamin B9 levels in quinoa (*Chenopodium quinoa*), and I abundance in the seaweed *Saccorhiza polyschides*, a functional hamburger rich in these nutrients by using these ingredients was developed. This research focused on the factors affecting its quality by examining the impact of cooking (steaming at 100 °C, roasting at 180 °C, grilling at 180 °C) and storage time (after 4 and 6 months at −20 °C) upon the product's properties.

Cooking treatments were found to influence the burger's colour and texture, whereas storage duration impacted FA levels and the polyene index. Cooked burgers presented lighter (L*, 45.1–55.0 *vs* 36.9 ± 2.4) and more yellow colouration (b*, 15.8–17.8 *vs* 13.6 ± 1.0) than raw burgers. Cooked burgers also exhibited higher textural values across various parameters than their raw versions. Grilled burgers (excluding initial time) were firmer (50.0 ± 5.1 N) than those cooked otherwise (37.0–39.9 N). Regarding FA levels, a decrease in DHA was recorded after four months (21.8–23.0% *vs* 26.4–30.6%). The polyene index followed a similar trajectory, declining from 2.6 to 3.6 initially to 1.8–1.9 in the fourth month. Hence, the studied mackerel burger could be a promising source of EPA, DHA, and other n-3 PUFAs in human diets, optimally with a frozen storage duration of fewer than four months to preserve nutritional integrity.

## Highlights

• Cooking affected colour & texture, whereas storage duration impacted FA levels.
• Grilled burgers were >25 % firmer than those cooked otherwise.
• DHA content decreased after 4 months of storage (21.8–23.0% *vs* 26.4–30.6%).
• The polyene index declined from 2.6 to 3.6 initially to 1.8–1.9 in the fourth month.

## Graphical abstract

**Figure:**
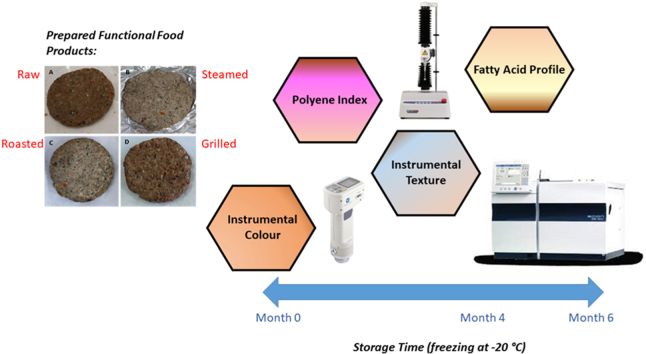

## 1 Introduction

A highly innovative functional fish burger for preventing cognitive ageing was previously developed and its key nutrients were determined in a previous experimental work [1]. These nutrients and compounds were targeted by a very specific formulation including chub mackerel (*Scomber colias*), quinoa (*Chenopodium quinoa*), and seaweed (*Saccorhiza polyschides*). In fact, due to these ingredients, high levels of docosahexaenoic acid (DHA, 22:6 n-3), a polyunsaturated fatty acid (n-3 PUFA), selenium (Se), and iodine (I) were achieved. According to animal studies, the regular intake of these components may be protective against neuronal disease [2]. Moreover, based on available evidence, the novel functional food aimed at potential synergies between DHA, other n-3 PUFAs, Se, and I [3].

Culinary procedures are associated with the loss of essential components and protein denaturation, thus entailing a nutritional loss risk, especially for a functional food rich in n-3 PUFAs [4,5]. These FAs show a strong tendency to oxidation when exposed to high temperatures (such as in grilling) or after long periods of storage encompassing several months, resulting in nutritional quality loss and off-flavours that taken together have a negative effect on the shelf life of any food containing n-3 PUFAs [6]. The formation of primary (hydroperoxides) and secondary oxidation products (aldehydes and ketones) from n-3 PUFAs is a main part of such oxidative reactions leading to alterations of odour and taste [7,8]. Such spoilage phenomena are also linked to negative effects on texture, colour, and various other dimensions of sensory quality [9]. Moreover, the particular way of ensuring high n-3 PUFA content in a product is also influential on storage stability [10]. For instance, it has been reported that baking and storage did not affect the stability of microencapsulated n-3 PUFA, not worsening the quality of the tested product, a functional bread [11]. The other ingredients in the product and the type and temperature of storage may also affect product deterioration [12]. All these aspects highlight the importance of conducting culinary treatment and storage stability studies on the novel functional fish burger.

Therefore, the goals of this experimental study were to assess the influence of alternative culinary treatments (steaming, roasting, and grilling) and frozen storage time on relevant properties of a recently developed functional hamburger for the prevention of cognitive ageing, with a specific focus on key fatty acids relevant to Alzheimer's disease prevention, polyene index reflecting oxidation levels, and instrumentally-measured texture and colour. The whole investigation aimed to verify whether a 6 month frozen storage at −20 °C of the hamburgers ensures their quality.

## 2 Materials and methods

### 2.1 Preparation of the functional hamburgers

Raw materials and fish hamburgers were prepared as previously described [1]. Main key ingredients were chub mackerel (*Scomber colias*), quinoa (*Chenopodium quinoa*), and freeze-dried seaweed (*Saccorhiza polyschides*). In particular, while chub mackerel was used raw after mincing (Grindomix GM 200, Dusseldorf, Germany), quinoa was previously placed in cold water (being the water changed five times within a period of 2 h), washed to remove saponins and phytic acid, and then boiled for 20 min to reduce soluble oxalate content. The remaining ingredients were weighed and minced together. Finally, all the ingredients were mixed into a homogeneous dough. This pulp was taken out and used to mould 75 g hamburgers (approximately 1.5 cm in thickness and 8.5 cm in diameter).

**Fig. 1:**
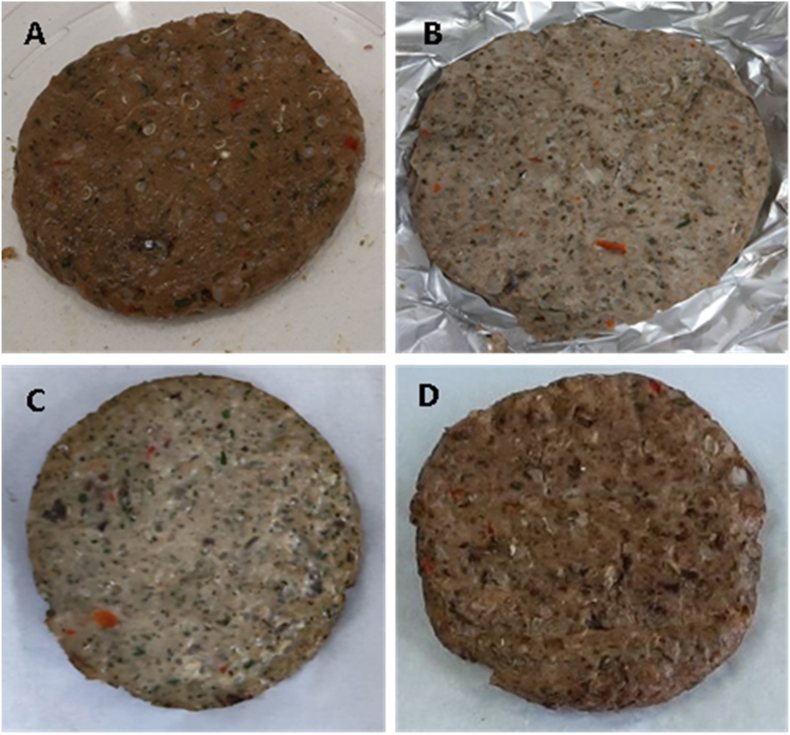
The prepared fish hamburgers (A: raw; B: steamed; C: roasted; and D: grilled).

Four sets of hamburgers (with nine hamburgers each) were separated and subjected to either no cooking procedure (raw) or to one of three culinary treatments: steaming, roasting, and grilling (Fig. 1 [A-D]). In the steaming treatment (Fig. 1B), each hamburger was wrapped in an aluminium foil and then subjected to cooking for 10 min with wet steam at 100 °C in a Rational CM6 oven (Rational, Landsberg am Lech, Germany). For roasting (Fig. 1C), the hamburger was also wrapped in an aluminium foil and then subjected to cooking for 10 min with dry heat at 180 °C in the same oven (Rational, Landsberg am Lech, Germany). Finally, grilling was done in a typical household griller (Flama Sketch, 230 V, 50 Hz, 2000 W) at approximately 180 °C (dry heat) for 10 min (5 min for each side of the hamburger) (Fig. 1D).

### 2.2 Storage of the functional hamburgers

All four sets of prepared hamburgers (raw, steamed, roasted, and grilled) were subjected to a storage stability study with exactly the same conditions. Indeed, after cooking, the hamburgers were stored at −20 °C during different periods (0, 4, and 6 months). For each of these three periods, three hamburgers (of a total of nine) were taken out from frozen storage for analysis, each set (raw, steamed, roasted, and grilled) being split in half into two sub-sets: one immediately analysed for the determination of textural and colour parameters (only for the cases of 0 and 6 months) and another analysed for fatty acid quantification after being frozen at −80 °C overnight, freeze-dried during 48 h at −45 °C, homogenized, and then left under vacuum at −80 °C (for all three time periods). Accordingly, each sub-set sample comprised three half burgers of 1.5 cm in thickness and 8.5 cm in diameter.

### 2.3 Instrumental colour determination

To determine the main colorimetric parameters of the prepared hamburgers (raw, steamed, roasted, and grilled), a portable colour meter, CR-400 Chroma Meter (Konica Minolta, Chiyoda, Japan), and a computer with specific software were used. Three measurements were taken on each side of each half hamburger, previously divided into two rectangular pieces measuring 2.5 cm × 2.5 cm × thickness (∼1.5 cm). Before measurements, the device was calibrated with a standard (CIELAB system: L*, 93.72; a*, −4.97; b*, 8.38). For the calculation of L* (lightness), a* (hue corresponding to a colour variation from green to red), and b* (hue corresponding to a colou variation from blue to yellow), the averages of the colour determinations on each side were used. In order to achieve a different appraisal of colour variation, L*, a*, and b* were used as inputs in formulas to calculate chroma (square root of the sum of squared a* and b*) and hue (arctangent of b*/a* in degrees) values.

### 2.4 Instrumental texture determination

The hamburgers were subjected to a texture profile analysis (TPA). Such TPA was done in a TA.XT Plus texturometer (SMS, Godalming, United Kingdom) coupled to a computer with specific texture analysis software. Prior to measurement, each half hamburger was divided into two portions and a rectangular piece with a size of 2.5 cm × 2.5 cm × thickness (∼1.5 cm) was excised from each portion. Then, each piece at a time was placed in the equipment and individually analysed, thereby enabling two readings per half burger and six readings in total for each sample. The TPA involved compression of the piece of hamburger on the texturometer's flat plate with a specific cylindrical probe (50 mm diameter) using a 30 kg load cell. Preliminary trials were made to define a compression threshold in order to avoid cracking in the analysed samples. Hence, it was defined that samples were to be compressed to 60 % of their original thickness by applying a constant compression speed of 1 mm/s. A set of six textural properties were evaluated: (i) hardness (in N); (ii) adhesiveness (in N); (iii) cohesiveness (adimensional); (iv) gumminess (in N); (v) springiness/sponginess (adimensional); (vi) and chewiness (in N).

### 2.5 Fatty acid profile

Methyl esters of the fatty acids (also known as FAME) were prepared from the freeze-dried experimental products (sampled at 0, 4, and 6-month periods) by acid-catalysed esterification using a previously described procedure [13]. The identification of the FAME was based on chromatographic retention time, using a standard (PUFA-3, Menhaden oil, Sigma-Aldrich) as reference. FAME content was calculated as a percentage of the total FAME amount. Analyses were always done in triplicate.

### 2.6 Calculation of the polyene index

To study the degree of lipid oxidation over storage time, the polyene index (PI) was determined. This index consists of the ratio between the FAs most susceptible to oxidation, as is the case of PUFA, especially those with a high number of double bonds, such as eicosapentaenoic acid (EPA, 20:5 n-3) and DHA, and the most stable FAs, as is the case of 16:0. The determination of this index was carried out according to the following formula [14]:

PI = ([EPA]+[DHA])/[16:0] Where,

[EPA] – Relative concentration of EPA in the total FAME (%);

[DHA] - Relative concentration of DHA in the total FAME (%);

[16:0] - Relative concentration of 16:0 in the total FAME (%).

### 2.7 Statistical analysis

The Kolmogorov-Smirnov's test and Levene's F-test were used to verify whether experimental data verified the normality and variance homogeneity assumptions, respectively. All tested data met these assumptions, thus enabling the application of parametric tests. Accordingly, data were analysed by factorial (considering interaction of culinary treatment and storage time) ANOVA distribution using the Tukey HSD post hoc test to determine the difference in the studied properties. In all tests, a value of 0.05 was used as significance level (α). All statistical treatment of data was done with the program STATISTICA 6 (Stat-sof, Inc., USA, 2003).

## 3 Results and discussion

### 3.1 Colour

The parameters of colour in the cases of raw and cooked (steamed, roasted, and grilled) hamburgers at the initial and final times of the storage period are shown in Table 1.

**Table 1:** Colour parameters (L*, a*, b*, Chroma, and Hue) of the raw and cooked chub mackerel hamburger products immediately after preparation and after 6-month storage.

Type of product	Storage time (month)	L*	a*	b*	Chroma	Hue (°)
Raw hamburger	0	36.9 ± 2.4^aA^	2.5 ± 0.8^aA^	13.6 ± 1.0^aA^	13.9 ± 0.9^aA^	79.5 ± 3.3^aA^
	6	36.9 ± 2.4^aA^	2.5 ± 0.8^aA^	13.6 ± 1.0^aA^	13.9 ± 0.9^aA^	79.5 ± 3.3^aA^
	p value	1.000	1.000	1.000	1.000	1.000
Steamed hamburger	0	53.6 ± 1.8^dA^	0.3 ± 1.2^bA^	16.7 ± 0.9^bA^	16.7 ± 0.9^bA^	89.1 ± 4.2^cA^
	6	55.0 ± 1.5^cA^	−0.1 ± 0.7^bA^	17.1 ± 1.5^bA^	17.1 ± 1.5^bA^	90.4 ± 2.4^bA^
	p value	0.907	0.991	0.997	0.998	0.993
Roasted hamburger	0	49.8 ± 2.7^cA^	1.1 ± 1.6^abA^	16.6 ± 1.8^bA^	16.7 ± 1.8^bA^	86.5 ± 5.2^bcA^
	6	52.6 ± 0.7^cA^	0.0 ± 0.4^bA^	16.2 ± 0.9^bA^	16.2 ± 0.9^bA^	90.0 ± 1.4^bA^
	p value	0.230	0.201	0.995	0.985	0.390
Grilled hamburger	0	45.1 ± 3.3^bA^	2.0 ± 0.8^aA^	15.8 ± 1.1^bA^	15.9 ± 1.0^bA^	82.9 ± 3.4^abA^
	6	47.1 ± 2.1^bA^	1.8 ± 0.6^aA^	17.8 ± 0.9^bB^	17.9 ± 1.0^bB^	84.4 ± 1.7^aA^
	p value	0.658	1.000	0.020	0.026	0.984

Values are presented as the mean ± standard deviation. In the same column, for the same storage time, different lowercase letters between types of hamburger represent significantly different arithmetic means (p < 0.05). In the same column, for each type of hamburger, different uppercase letters represent significantly different arithmetic means (p < 0.05) between the beginning and the end of the storage period.

The first and large contrast was observed between the colour parameters of raw and cooked hamburgers. As already visible in the photos, raw fish burgers (Fig. 1A) were much darker than the other ones, 36.9 ± 2.4 *vs* 45.1–55.0 (p < 0.001). Regarding the other colour parameters, while contrast was weakened for a* and hue, it was also strong for b* and chroma. Indeed, a stronger yellow hue was registered in the cooked product, 15.8–17.8 *vs* 13.6 ± 1.0 (raw product), p = 0.01. Among cooked burgers, the steamed product was lighter than the others (53.6–55.0 *vs* 45.1–52.6, p = 0.03) and less red than the grilled one (−0.1 to 0.3 *vs* 1.8 to 2.0, p = 0.04). Grilled fish burgers were darker than the roasted ones, 45.1–47.1 *vs* 49.8–52.6 (p = 0.002), but differences were almost non-existing for the other parameters. The effect of storage time on instrumental colour was very weak, being only detected in the case of grilled fish burgers, which became more yellow after 6-month storage, 17.8 ± 0.9 *vs* 15.8 ± 1.1 (p = 0.02). The variation of the chroma parameter reflected the variation of b* in this case. For all other cases, no difference was observed due to storage time.

Different authors [[15], [16], [17]] have prepared fish burgers and measured their colour, but with different CIELAB space results. For instance, for tilapia (*Oreochromis niloticus*) burgers [15], L* values were higher than in the current study with mackerel burgers, thereby ranging from 69.1 ± 1.0 in uncooked to 63.9–65.5 in cooked products. Similar L* values were attained in other experimental work [17], but for raw fish burgers made from the surimi paste of black tilapia and potato flour. For some cooked burgers, this study reported an L* of 52.4–54.8, which overlaps with values measured in steamed and roasted mackerel burgers. Other researchers found much lower L* values, ranging in the 33.3–40.6 interval [16]. These authors observed a depression in lightness due to the incorporation of 1 %, w/w, dry seaweed in common barbel (*Barbus barbus*) burgers. This may be a reason for depressing L* in the mackerel burgers, since they were enriched with 0.5 % freeze-dried brown seaweed (*S. polyschides*). In this regard, it was proved that fucoxanthin —a carotenoid in brown seaweed— reduced the lightness value [18]. The b* values were also higher in the tilapia burgers [15], but the hue angle values were closer to those in mackerel burgers.

The effect of cooking on burger colour and, particularly, on L* may result of the intense denaturation of chub mackerel muscle protein that occurs by applying a thermal treatment [19,20]. This protein denaturation lowers porosity in the product's structure and increases opacity, thereby enhancing light reflection. In particular, it is known that opacity increases when the internal temperature is in the 45–67 °C interval, as denaturation of myosin and actin, which do not contribute to a red colour, overrides the red colour associated with myoglobin [21,22]. Similar observations have been done for other equivalent products [19].

Moreover, myoglobin protein contains the primary heme pigment responsible for a meaty colour and heating has a strong effect on this protein, affecting a*, b*, and hue [19]. A decrease of a* and an increase of the hue angle values upon cooking has been observed in other types of hamburgers [19]. Such colour changes have been explained by interconversion of three forms of myoglobin and their degradation through redox reactions [23]. The denaturation of myosin and actin may also lead to pigmentation that alters the original red colour associated with myoglobin [21,22]. Furthermore, an enhancement of the yellowness (b*) parameter after cooking was previously observed by other researchers [15,24]. Just as in mackerel burgers, these authors [15,24] also observed an approximation of the colour of cooked fish burgers to the yellow axis (hue angle increasing and nearing 90°) with cooking. Specifically concerning grilling, observations similar to the current study have been made [15]: darker grilled fish burgers with a reddish colour (higher a* value) in comparison to the roasted fish burgers were observed. Such colour differences were ascribed to the intense contact of the burger's surface with the grilling device grids [15]. The high local temperatures intensify the occurrence of non-enzymatic browning reactions, which always occur in products subjected to thermal treatment, such as the caramelization and Maillard reactions [25].

The strong stability of colour parameters over frozen storage time suggests that product quality did not decay even after 6 months and proves that burger appearance was largely unchanged. This is highly relevant, since colour is one of the key parameters that determine consumer acceptance of food [16,26]. For other muscle-based raw and processed products frozen stored for up to 6 months, a similar absence of substantial colour change over storage time has been found [27]. However, other authors [28] have also reported increased lightness values after freezing due to denaturation of protein, but this study was mainly focused on the influence of variable freeze-thaw cycles on broiler meat's quality. In the case of the fish burger, it seems that any biochemical change that occurred in the intervening period did not denature protein to the extent of altering the microstructure of the products and that frozen storage at −20 °C is a viable solution to prevent colour changes.

### 3.2 Texture

The instrumental texture parameters of the raw and cooked (steamed, roasted, and grilled) hamburgers at the initial and final times of the storage period are presented in Table 2.

**Table 2:** Texture parameters (hardness, adhesiveness, cohesiveness, gumminess, sponginess, and chewiness) of the raw and cooked chub mackerel hamburger products immediately after preparation and after 6-month storage.

Type of product	Storage time (month)	Hardness (N)	Adhesiveness (N)	Cohesiveness	Gumminess (N)	Sponginess	Chewiness (N)
Raw hamburger	0	4.3 ± 0.9^aA^	−1.3 ± 0.1^aA^	0.58 ± 0.04^aA^	2.5 ± 0.4^aA^	0.72 ± 0.05^aA^	1.8 ± 0.4^aA^
	6	7.5 ± 1.4^aA^	−0.7 ± 0.2^aB^	0.53 ± 0.01^aA^	4.0 ± 0.7^aA^	0.60 ± 0.02^aB^	2.4 ± 0.3^aA^
	p value	0.947	0.000	0.169	0.996	0.000	1.000
Steamed hamburger	0	41.6 ± 3.4^bcA^	0.0 ± 0.0^bA^	0.61 ± 0.05^bA^	25.4 ± 1.3^bA^	0.82 ± 0.01^bA^	20.7 ± 0.9^bA^
	6	39.9 ± 0.2^bA^	−0.2 ± 0.1^bA^	0.67 ± 0.01^bA^	26.8 ± 0.5^bcA^	0.85 ± 0.03^bA^	22.7 ± 0.7^bcA^
	p value	0.999	0.895	0.052	0.996	0.759	0.971
Roasted hamburger	0	48.6 ± 4.5^cdA^	0.0 ± 0.0^bA^	0.60 ± 0.03^bA^	29.2 ± 2.9^bA^	0.85 ± 0.02^bA^	24.9 ± 2.8^bA^
	6	37.0 ± 0.9^bB^	−0.4 ± 0.4^bB^	0.67 ± 0.01^bB^	24.7 ± 0.3^bA^	0.85 ± 0.03^bA^	21.0 ± 0.5^bA^
	p value	0.010	0.036	0.026	0.413	1.000	0.547
Grilled hamburger	0	52.9 ± 8.5^dA^	0.0 ± 0.0^bA^	0.71 ± 0.03^cA^	37.3 ± 6.6^cA^	0.85 ± 0.03^bA^	31.9 ± 6.5^cA^
	6	50.0 ± 5.1^cA^	−0.1 ± 0.1^bA^	0.67 ± 0.02^bA^	33.5 ± 3.9^cA^	0.83 ± 0.01^bA^	27.8 ± 3.5^cA^
	p value	0.972	0.930	0.524	0.596	0.912	0.456

Values are presented as the mean ± standard deviation. In the same column, for the same storage time, different lowercase letters between types of hamburger represent significantly different arithmetic means (p < 0.05). In the same column, for each type of hamburger, different uppercase letters represent significantly different arithmetic means (p < 0.05) between the beginning and the end of the storage period.

The results can be analysed along with the culinary treatment and storage time effects on the studied textural parameters. For the former effect, as in the case of instrumental colour (see previous section), the starkest opposition was registered between raw and cooked fish burgers. Indeed, for all textural parameters, raw burgers exhibited lower values than cooked burgers (p < 0.001). However, considering adhesiveness in modulus, raw hamburgers had a higher adhesiveness than the other ones, 0.7–1.3 N *vs* 0.0–0.4 N (p < 0.001). The studied raw functional food was also much softer, less cohesive, and spongy than the cooked product regardless of the particular culinary treatment with 4.3–7.5 N *vs* 37.0–52.9 N (p < 0.001), 0.53–0.58 *vs* 0.61–0.71 (p = 0.02), and 0.60–0.72 *vs* 0.82–0.85 (p < 0.001), respectively. The values of gumminess as the product of hardness and cohesiveness and chewiness as the product of gumminess and sponginess necessarily depicted a similar raw-cooked contrast. Among cooked hamburgers, the grilled product had a TPA that was more differentiated from the other ones. In fact, except initial sampling, grilled fish burgers were harder 50.0 ± 5.1 N *vs* 37.0–39.9 N than the others (p = 0.03) and, if the final sampling is excluded, displayed higher gumminess and chewiness, 37.3 ± 6.6 N *vs* 25.4–29.2 N (p = 0.01) and 31.9 ± 6.5 N *vs* 20.7–24.9 N (p = 0.03), respectively. In the cases of cohesiveness and sponginess, almost no difference was produced by the various applied culinary treatments. On the other hand, the storage time effect on texture was confined to the raw and roasted burgers. In the former, there was a loss of adhesiveness (in modulus) and sponginess —a very substantial loss from 0.72 ± 0.05 to 0.60 ± 0.02 (p < 0.001)— over storage time. In the latter, roasted burger, a softening after 6-month storage, 37.0 ± 0.9 N *vs* 48.6 ± 4.5 N (p = 0.01), was coupled with an increment of cohesiveness, 0.67 ± 0.01 *vs* 0.60 ± 0.03 (p = 0.026).

The instrumental texture of fish burgers has not been the subject of many studies [[15], [16], [17],^25^,^29^,^30^]. Another problem derives from the wide variability of types of burgers and measurement conditions that make comparisons between studies particularly difficult. Hardness, gumminess, and chewiness levels highly depend on the degree of compression, being higher with stronger compression. For other textural parameters, the comparison is also not straightforward. Cohesiveness levels between 0.62 and 0.84 for tilapia surimi paste burgers have been measured [17], which are relatively similar to those values for mackerel burgers. The results attained with tilapia burgers [25]—60.1–62.1 N hardness, 0.72–0.75 cohesiveness, and 43.7–46.6 N gumminess— were higher than in chub mackerel burgers, but still in the same order of magnitude. In the case of pacu (*Piaractus brachypomus*) burgers, textural hardness (22.1–48.1 N), cohesiveness (0.60–0.70), sponginess (0.81–0.86), and chewiness (13.2–23.4 N) were measured [30], being very similar to chub mackerel burgers, but with a 50 % compression in the TPA of the pacu burgers.

In the comparison of raw *vs* cooked burgers, no large increase in hardness, cohesiveness, and other textural parameters was observed by other researchers after cooking [17], thus deviating from burger hardening observations in the mackerel products. However, such hardening with cooking is expected, since the loss of water during cooking tends to increase hardness, chewiness, sponginess, and cohesiveness [31]. Other authors declared the existence of an inverse proportionality between moisture and hardness [32] that may explain the higher hardness measured in the grilled mackerel burgers, since these products contained 64.0 ± 0.2 %, w/w, moisture *vs* 67.8–70.3 %, w/w, in steamed and roasted mackerel burgers.

Regarding the storage time effect on fish burger texture, a progressive hardening of salmon (*Salmo salar*) and striped catfish (*Pangasius hypophthalmus*) hamburgers during 2-month frozen storage at −18 °C has been registered [33]. This finding contrasts with the absence of any hardening in mackerel burgers even after 6 months of storage at a similar temperature (−20 °C). Such textural phenomenon has also been observed in the same type of product by other researchers, but it should be noted that in this case it involved accelerated shelf-life testing with incubation at 25, 35, and 45 °C for 3 months [34]. Other researchers also reported the hardening of tilapia fish burgers after 1-month storage, but no further change occurred in the following 5 months [35]. In the case of salmon and catfish burgers [33], there was no evolution in cohesiveness and chewiness over storage time, thus agreeing with the present study's results. In any case, the absence of any trend in key textural parameters has been observed in other similar experimental works, for instance in a modelling study of frozen beef burgers [36]. In this regard, it should be noted that freezing reduces microbial and enzymatic activity, thus better preserving properties than chilled storage [37]. However, ice crystal formation during freezing is a problem: the larger the ice crystals are formed, the higher is the probability of texture alteration [37]. The faster and less heterogeneous the freezing occurs, the smaller and more uniform the ice crystals will be [38,39]. Once freezing has occurred, it is also crucial to keep the ice crystals small. This can be achieved by a very stable storage temperature, as thawing and refreezing and temperature oscillations generate larger ice crystals [38,39]. The results of the current study on fish burgers seem to show that proper care was taken in freezing quickly and ensuring a nearly constant frozen storage temperature.

The texture is indeed another key set of properties that determine consumer acceptance of food products. The high percentage of chub mackerel in the formulation (>72 %, w/w) seems to have been advantageous to the developed functional food, namely, in ensuring firmness and cohesiveness. Indeed, fish proteins display a set of exceptional functional properties, comprising gelling properties [40], and minced fish has successfully been used for conferring cohesion–adhesion, succulence, and chewiness to different foods [41]. This fact contributed to a TPA —even after 6-month storage— that is in the same order of magnitude (or above) as that reported for usual beef burgers [42].

### 3.3 Fatty acids and polyene index

The FA relative contents (% of total FA), n-3/n-6 ratio, and the polyene index of studied fish burgers are displayed in Table 3.

**Table 3:** Polyene index and main lipid fraction parameters (%) used in its calculation as well as main fatty acid classes (%) and n-3/n-6 ratio of raw and cooked chub mackerel hamburger products immediately after preparation and after 4- and 6-month storage.

Type of product	Storage time (month)	16:0 (%)	20:5 n-3 (%)	22:6 n-3 (%)	Polyene Index	SFA (%)	MUFA (%)	PUFA (%)	n-3/n-6
Raw hamburger	0	12.2 ± 0.9^aA^	9.8 ± 0.2^aA^	29.3 ± 2.2^aA^	3.2 ± 0.4^aA^	22.7 ± 1.1^aA^	21.9 ± 1.3^aA^	53.2 ± 2.5^aA^	6.6 ± 0.2^abA^
	4	16.3 ± 0.2^aB^	8.1 ± 0.0^aB^	22.9 ± 0.3^aB^	1.9 ± 0.1^aB^	29.0 ± 0.4^aB^	24.7 ± 0.2^aB^	43.9 ± 0.5^aB^	2.4 ± 0.0^aB^
	6	16.4 ± 0.4^aB^	8.1 ± 0.2^aB^	22.4 ± 0.6^aB^	1.9 ± 0.1^aB^	29.2 ± 0.7^aB^	24.7 ± 0.4^aB^	43.5 ± 1.0^aB^	2.4 ± 0.0^aB^
	p value	0.019	0.035	0.020	0.019	0.021	0.029	0.022	0.010
Steamed hamburger	0	13.6 ± 0.3^bA^	9.3 ± 0.1^bA^	26.4 ± 0.3^bA^	2.6 ± 0.1^bA^	24.8 ± 0.5^aA^	23.1 ± 0.0^aA^	49.5 ± 0.5^aA^	6.4 ± 0.1^aA^
	4	16.4 ± 0.6^aB^	7.9 ± 0.1^aB^	22.6 ± 0.8^aB^	1.9 ± 0.1^aB^	29.3 ± 0.6^aB^	25.1 ± 0.2^aB^	43.4 ± 1.0^aB^	2.4 ± 0.0^aB^
	6	16.5 ± 0.3^aB^	8.1 ± 0.0^aB^	22.4 ± 0.3^aB^	1.9 ± 0.1^aB^	29.3 ± 0.4^aB^	24.9 ± 0.2^aB^	43.2 ± 0.5^aB^	2.5 ± 0.0^aB^
	p value	0.025	0.028	0.023	0.014	0.018	0.022	0.028	0.011
Roasted hamburger	0	11.6 ± 0.5^aA^	9.6 ± 0.1^abA^	30.0 ± 0.4^aA^	3.4 ± 0.2^aA^	22.0 ± 0.6^aA^	22.1 ± 0.2^aA^	53.9 ± 0.3^aA^	6.7 ± 0.1^bA^
	4	16.9 ± 0.5^aB^	7.8 ± 0.2^aB^	21.8 ± 0.8^aB^	1.8 ± 0.1^aB^	30.0 ± 0.8^aB^	25.0 ± 0.2^aB^	42.3 ± 1.1^aB^	2.4 ± 0.0^aB^
	6	16.6 ± 0.3^aB^	8.0 ± 0.1^aB^	22.5 ± 0.4^aB^	1.8 ± 0.1^aB^	29.5 ± 0.5^aB^	25.0 ± 0.1^aB^	43.2 ± 0.6^aB^	2.4 ± 0.0^aB^
	p value	0.018	0.038	0.020	0.013	0.018	0.017	0.022	0.010
Grilled hamburger	0	11.3 ± 0.4^aA^	9.9 ± 0.1^aA^	30.6 ± 0.7^aA^	3.6 ± 0.2^aA^	21.6 ± 0.6^aA^	21.6 ± 0.2^aA^	54.7 ± 0.7^aA^	6.8 ± 0.2^bA^
	4	16.3 ± 0.3^aB^	8.1 ± 0.1^aB^	23.0 ± 0.2^aB^	1.9 ± 0.1^aB^	27.6 ± 2.2^aB^	24.0 ± 1.2^aB^	45.8 ± 3.2^aB^	2.4 ± 0.0^aB^
	6	16.5 ± 0.5^aB^	8.0 ± 0.1^aB^	22.3 ± 0.3^aB^	1.8 ± 0.1^aB^	28.2 ± 2.6^aB^	24.2 ± 1.4^aB^	45.1 ± 3.8^aB^	2.4 ± 0.1^aB^
	p value	0.017	0.032	0.019	0.015	0.041	0.023	0.039	0.012

Values are presented as the mean ± standard deviation. SFA – Saturated fatty acids. MUFA – Monounsaturated fatty acids. PUFA – Polyunsaturated fatty acids. In the same column, for the same storage time, different lowercase letters between types of hamburger represent significantly different arithmetic means (p < 0.05). In the same column, for each type of hamburger, different uppercase letters represent significantly different arithmetic means (p < 0.05) over storage time.

Firstly, concerning the effect of the culinary treatment on the selected FA contents, most FAs or major FA groups (SFA, MUFA, PUFA) did not present statistical differences. Almost no difference was detected in the case of the n-3/n-6 ratio. In this case, there was only a slight contrast between the steamed fish burger and the other cooked burgers at the beginning of the storage experiment, 6.4 ± 0.1 *vs* 6.7–6.8 (p = 0.01). Steamed hamburgers were less rich in two highly relevant n-3 PUFAs than the other hamburgers: 9.3 ± 0.1 % *vs* 9.8–9.9 % for EPA (p = 0.02, with exception of the roasted hamburger) and 26.4 ± 0.3 % *vs* 29.3–30.6 % for DHA (p = 0.01), respectively. For total SFA, MUFA, and PUFA, culinary treatment did not play any meaningful role. The polyene index, by its formula, reflects the lower DHA contents in the initial steamed fish burger, thereby yielding a lower index in comparison to the other burgers, 2.6 ± 0.1 *vs* 3.2–3.6 (p = 0.01). Apart from the culinary effect, storage time was highly influential on FA contents and the polyene index. After 4-month storage there was an increase in the palmitic acid level (from 11.3-13.6 % to 16.3–16.9 %, p = 0.005) and a reduction in EPA and DHA levels, from 9.3-9.9 % to 7.8–8.1 % (p = 0.02) and from 26.4-30.6 % to 21.8–23.0 % (p = 0.01), respectively. The levels after 4 months did not vary further after two additional frozen storage months (p = 0.35). Moreover, the total SFA, MUFA, and PUFA levels also varied after 4-month storage (p = 0.03), but remained unchanged afterwards. Whereas SFA and MUFA levels at months 4 and 6 were higher than at the beginning of the experiment, the opposite occurred in the case of total PUFA content. The n-3/n-6 ratio registered a strong variation, declining from 6.4 to 6.8 at month 0 to 2.4 after 4-month storage (p = 0.01) and remaining approximately at this level (2.4–2.5) after 6-month storage. Finally, the polyene index also declined from 2.6 to 3.6 at the initial time point to 1.8–1.9 at the fourth month (p = 0.01) and, similarly, 1.8–1.9 at the end of the experiment.

Firstly, it must be referred that culinary treatment did not strongly affect the FA levels. Hence, cooking, even in its more thermally drastic form (grilling), did not decompose the most thermo-sensitive FA, the PUFA, especially those with a higher number of double bonds that are more prone to chemical alterations [43]. The influence of temperature on the FA profile is assessed differently by several research groups [[44], [45], [46], [47]]. This disagreement may result from diverse temperature-time combinations for the same type of culinary treatment and species-specific phenomena. Indeed, there are research groups reporting a negative effect, especially of grilling, on EPA, DHA, and other PUFA levels [44,46]. Other groups did not observe any effect [47], concluding that baking, boiling, and grilling did not alter the FA composition of the catfish fillets.

Concerning the storage time effect on the FA composition, the reduction of EPA, DHA, and PUFA contents as well as the decline of the n-3/n-6 and polyene index in raw and all types of cooked hamburgers indicate substantial FA degradation phenomena in the first 4 months of storage. These phenomena may be linked to oxidation reactions, such as those that have been reported in catfish and salmon burgers and measured by oxidation indicators [34]. For instance, thiobarbituric acid (TBA) value is a major indicator of lipid oxidation as it correlates with the malondialdehyde concentration, a secondary product of lipid oxidation of unsaturated FAs [48]. A more intense oxidation with higher n-3 PUFA levels in burgers with a higher proportion of salmon, a fatty fish species, has been observed [34]. This may also be the case for the mackerel burgers, since they are very rich in n-3 PUFA, containing 35–40 % of EPA and DHA. Similar increases in palmitic acid, SFA, and MUFA levels with concomitant declines in EPA, DHA, and PUFA levels have also been reported for anchovy (*Engraulis encrasicolus*) burgers stored at −18 °C for 4 months [49]. It also agrees with a study on FA in frozen sardine (*Sardinella aurita*) meat [50]. These FA level variations are ascribed to the decomposition of PUFAs, thereby generating compounds with low molecular weight and probably short-chain FAs. A long hydrocarbon chain and a high degree of unsaturation in PUFA make them more prone to reactions of hydrolysis and processes of oxidation than the SFA [51]. Indeed, the PUFA/SFA ratio has been reported to decrease with frozen storage [49]. Other authors also observed a decline in this ratio during a 3-month storage of seafood at −22 °C [6]. This has led to the proposal of the polyene index as an adequate index for monitoring lipid oxidation [52]. It may be concluded that this index is an appropriate parameter for determining the level of oxidative rancidity in fish burgers [49]—a conclusion also corroborated in the case of mackerel burgers kept in frozen storage. Finally, it is also worth noting that this observed degradation of nutritional quality over storage time did not meaningfully impact colour and texture, the two main aspects of the sensory quality of mackerel burgers that were studied.

### 3.4 Storage stability overall assessment

The various determined aspects of product quality over storage time, encompassing colour, texture, fatty acid profile, and oxidation level, can be combined into a global assessment of the storage stability of the fish hamburger. The first and foremost issue is the concentration of EPA and DHA — which, together with other nutrients, can be considered as bioactive compounds preventing and/or delaying cognitive ageing and related pathologies [2]— in the functional food after 4- and 6-month storage. In the absence of established dosages for an effect of EPA and DHA on cognitive ageing, it may be considered the human EPA + DHA requirements according to the EFSA Panel on Dietetic Products, Nutrition and Allergies, which set the Dietary Reference Values (DRV) for the EU population [53], corresponding to a daily Adequate Intake (AI) of 250 mg EPA + DHA. After 4-month storage and considering lipid content variation with culinary treatment —between 5.1 %, w/w, in raw and 6.7 %, w/w, in grilled hamburgers—, the AI could be ensured with 22.5 g raw, 20.7 g steamed, 18.6 g roasted, and 17.1 g grilled hamburger. Furthermore, after 6-month storage, the AI would require 22.8 g raw, 20.7 g steamed, 18.0 g roasted, and 17.6 g grilled hamburger. These are still perfectly viable amounts, though, ideally, the initial functional hamburger would be the best source of EPA and DHA (with only 15.3 g raw, 15.0 g steamed, 12.3 g roasted, and 12.1 g grilled hamburger). The other parameters of quality seemed to withstand the long storage time and indicate that consumer acceptance would not be a major hurdle. Hence, the fish burger can be a suitable source of EPA and DHA, providing a relevant contribution to health and wellness of consumers. This enables to consider chub mackerel a nutraceutical ingredient and the prepared burger a functional food [54], provided that consumption would happen ideally less than after 4 months of frozen storage.

## 4 Conclusions

Cooking processes and different culinary treatments introduce significant colour and textural variances in mackerel burgers, whereas storage duration primarily influences key fatty acid (FA) levels and the polyene index. However, results showed that nutritional quality deterioration over storage time does not correspond with changes in colour, texture, and sensory quality in the mackerel burgers. In any case, even after 4-month storage, the daily EPA and DHA requirements could be met with only 22.5 g raw, 20.7 g steamed, 18.6 g roasted, and 17.1 g grilled hamburger. Due to moisture loss, the grilled product had a higher nutritional density. In summary, this seaweed-fortified mackerel burger could be a promising source of EPA, DHA, and other n-3 PUFAs in human diets, optimally with a frozen storage duration of fewer than four months to preserve nutritional integrity.

## Ethics statement

There are no ethics issues.

## Data availability statement

Has data associated with your study been deposited into a publicly available repository? No. Data will be made available on request.

## CRediT authorship contribution statement

**J. Valentim:** Methodology, Investigation, Conceptualization. **C. Afonso:** Writing – review & editing, Conceptualization. **R. Gomes:** Methodology, Investigation. **A. Gomes-Bispo:** Methodology, Investigation. **Prates J.A.M.:** Writing – review & editing, Supervision. **N.M. Bandarra:** Writing – review & editing, Supervision. **C. Cardoso:** Writing – review & editing, Writing – original draft.

## Declaration of competing interest

The authors declare that they have no known competing financial interests or personal relationships that could have appeared to influence the work reported in this paper.
